# High-Dose Cytarabine in Acute Myeloid Leukemia Treatment: A Systematic Review and Meta-Analysis

**DOI:** 10.1371/journal.pone.0110153

**Published:** 2014-10-09

**Authors:** Wei Li, Xiaoyuan Gong, Mingyuan Sun, Xingli Zhao, Benfa Gong, Hui Wei, Yingchang Mi, Jianxiang Wang

**Affiliations:** 1 Leukemia Diagnosis and Treatment Center, Institute of Hematology and Blood Disease Hospital, Chinese Academy of Medical Sciences and Peking Union of Medical College, Tianjin, China; 2 State Key Laboratory of Experimental Hematology, Institute of Hematology and Blood Disease Hospital, Chinese Academy of Medical Sciences and Peking Union of Medical College, Tianjin, China; Emory University/Georgia Institute of Technology, United States of America

## Abstract

The optimal dose, scheme, and clinical setting for Ara-C in acute myeloid leukemia (AML) treatment remain uncertain. In this study, we performed a meta-analysis to systematically assess the impact of high-dose cytarabine (HDAC) on AML therapy during the induction and consolidation stages. Twenty-two trials with a total of 5,945 *de* *novo* AML patients were included in the meta-analysis. Only patients less than 60 year-old were included in the study. Using HDAC in induction therapy was beneficial for RFS (HR = 0.57; 95% CI, 0.35–0.93; *P* = 0.02) but not so for CR rate (HR = 1.01; 95% CI, 0.93–1.09; *P* = 0.88) and OS (HR = 0.83; 95% CI, 0.66–1.03; *P* = 0.1). In consolidation therapy, HDAC showed significant RFS benefits (HR = 0.67; 95% CI, 0.49–0.9; *P* = 0.008) especially for the favorable-risk group (HR = 0.38; 95% CI, 0.21–0.69; *P* = 0.001) compared with SDAC (standard dose cytarabine), although no OS advantage was observed (HR = 0.84; 95% CI, 0.55–1.27; *P* = 0.41). HDAC treatment seemed less effective than auto-BMT/allo-BMT treatment (HR = 1.66, 95% CI, 1.3–2.14; *P*<0.0001) with similar OS. HDAC treatment led to lower relapse rate in induction and consolidation therapy than SDAC treatment, especially for the favorable-risk group. Auto-BMT/allo-BMT was more beneficial in prolonging RFS than HDAC.

## Introduction

Cytarabine (Ara-C) has been a major drug for acute myeloid leukemia (AML) treatment for more than three decades. Initially, the drug was used at 100–200 mg/m^2^ for 7–10 days for standard treatment [1]. In recent years, multiple cycles of high-dose cytarabine (HDAC) therapy (at 3.0 g/m^2^ every 12 hours) have been commonly used as the consolidation therapy in multicenter trials; it was observed to maximize Ara-C’s anti-leukemia effect in AML patients, leading to improve disease- free-survival (DFS) [2], [3]. After that, HDAC instead of standard-dose cytarabine multiagent chemotherapy has become a common practice in the treatment of AML, especially in patients younger than 60 years of age, either for remission induction or consolidation, based on the guidelines of the National Comprehensive Cancer Network (*VI*. 2013). However, recent randomized controlled trials with 781 patients have challenged the benefits of HDAC [4]. HDAC failed to show significant improvement in five-year relapse-free survival and five-year overall survival as compared with SDAC regimen in AML treatments, especially in the consolidation therapy. After these new studies, the dose and effect of HDAC during AML induction and consolidation therapies are open for new evaluation [5]. Therefore, a systematic analysis needs to be performed to clarify these issues, which is the focus of this meta-analytical review. Specifically, this review study compared the effectiveness of HDAC versus SDAC as AML therapy in adult patients during the induction and consolidation phases, in order to shed lights on defining the optimal dose and scheme of Ara-C treatment with minimum possible toxicity. On the other hand, we assessed the effectiveness of HDAC compared with bone marrow transplantation (BMT) in order to explore the best therapy in the consolidation phase.

## Methods

### Literature Search

Independent reviewers (L.W and G. XY) systematically searched PubMed for relevant research papers published in English between January 1990 and March 2013 using the following query terms: acute myeloid leukemia, high-dose, and cytarabine. The titles and abstracts of the identified studies were reviewed to determine potential eligibility for meta-analysis. Relevant review and meta-analysis articles were included to identify additional studies that met the inclusion criteria. Further studies were referred by means of manual search of secondary sources. Divergences among the reviewers must be resolved to reach a consensus after further discussion.

### Inclusion and Exclusion Criteria

Identified articles were independently appraised according to the inclusion criteria by the same two reviewers (L.W and G. XY). All patients were required to have untreated acute myeloid leukemia, de novo AML, and patients with acute promyelocytic leukemia and translocation t(15;17) did not included this study. The included trials described the comparsion of HDAC (2.0–3.0 g/m^2^) and standard-dose cytarabine (SDAC, ≤200 mg/m^2^) in induction and consolidation therapy, or bone marrow transplantion (BMT) in consolidation therapy. new medicine research and phase II/III clinical trials were excluded. Studies reported hazard ratios (HRs) and 95% confidence intervals (CIs) for overall survival (OS)/relapse free survival (RFS) benefit, or those provided data to estimate HR by the method of Parmar *et al* [6]. If multiple articles were identified to report on the same study, the most recent one was analyzed. Only randomized controlled trials (RCT) were included in the comparison of HDAC and SDAC, but observed study met the inclusion criteria for the study of BMT vs HDAC, because it was difficult to ensure that each patient had donor and even more difficult to complete RCT.

**Table 1:** Characteristics of included Studies for induction therapy.

Source	StudyID	EnrollmentPeriod	Multi-center	No. ofpatients	RCTs	Study entrycriteria	Induction therapy
J.P. Matthewset al, 2001 [7]	ALSG	1987–1991	Yes	248	Yes	de novo AMLmedian age: 42 years	DEA (DNR+VP-16+Ara-c 100 mg/m^2^/d×7d)DEA (DNR+VP-16+Ara-c 3 g/m^2^ q12 h d1,3,5,7d)
JK. Weicket al, 1996 [8]	SWOG	1986–1991	Yes	723	Yes	de novo or secondaryAML M/F: 397/326 agerange: 15–64 years WBCrange: 0.4–416×10^9^/L	DA (DNR+Ara-c 200 mg/m^2^/d×7d)DA (DNR+Ara-c 3 g/m^2^ q12 h×6d)
T. Büchneret al, 2006 [9]▴	----	1999–2005	Yes	1770	Yes	de novo or secondaryAML, age range: 16∼85years 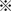	Double inductionTAD (6-TG+DNR+Ara-c 100 mg/m^2^/d×8d) + HAM (MTZ+Ara-c 3 g/m^2^ q12 h×3d)HAM+HAM (MTZ+Ara-c 3 g/m^2^ q12 h×3d)
T. Büchneret al, 1999 [10]	CAMLCG	1985–1992	Yes	725	Yes	de novo AML,M/F: 336/389 medianage: 44 (16–60) yearsWBC range: 0.1–405×10^9^/L	Double inductionTAD+TAD (6-TG+DNR+Ara-c100 mg/m^2^/d×8d)TAD (6-TG+DNR+Ara-c 100 mg/m^2^/d×8d) + HAM (MTZ+Ara-c 3 g/m^2^ q12 h×3d)
T. Büchneret al, 2009 [11]▴	CAMLCG	1993–2005	Yes	1284	Yes	de novo AML,years range: 16∼85 years 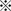 WBC range: 0.05–1017×10^9^/L	Double inductionTAD (6-TG+DNR+Ara-c 100 mg/m^2^/d×8d) + HAM (MTZ+Ara-c 3 g/m^2^ q12 h×3d)HAM+HAM (MTZ+Ara-c 3 g/m^2^ q12 h×3d)

Note: ▴ T. Büchner et al, 2009 repeated the same trial of T. Büchner et al, 2006.

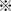
analyze <60 years patients in each trial.

Abbreviations: NR, not reported; IDA, idarubicin; Ara-c, cytarabin; VP-16, etoposide; DNR, daunorubicin.

#### Data Extraction

Data were extracted in the standardized format by two independent reviewers. Data collected for each study included study name, name of first author, year of publication, period of enrollment, total number of subjects allocated to therapies, median patient age (years), chemotherapy regimens, number of events (death, relapse) in each group, and study end points of overall survival benefit, RFS benefit, or both. We used overall survival (OS) and relapse free survival (RFS) of individual studies. Discrepancies in data extraction were resolved by identifying consensus, referring back to the original article, or contacting study authors if necessary. When missing data were encountered, the authors were contacted to complete data analysis.

#### Assessment of methodological quality

Two reviewers assessed the methodological quality of each trial. The risk of bias in each trial was assessed according to Cochrane methodology by using the following criteria: considering random sequence generation, allocation concealment, the blinding of patients and personnel, incomplete outcome data, selective reporting, and Begg’s funnel plots and Egger’s test were used to reveal possible publication bias. Heterogeneity was assessed by forest plots and with a standard Chi^2^ test and an inconsistency (*I^2^*) statistic. Both the fixed-effect model and the random-effect model were initially used to calculate total HRs, and finally selected with regards to heterogeneity in the survival analyses. If the heterogeneity (*I^2^*>75%) was too great for a summary estimate to be calculated, subgroup analysis was needed.

#### Data synthesis

Data were synthesized using the Cochrane Statistics package RevMan (version 4.0.4). The threshold of significance was *P*≤0.05. A forest plot with combined HRs (with 95% CIs) for OS and RFS benefit of SDAC *vs.* HDAC, or HDAC *vs*. auto-BMT/allo-BMT was constructed using random-effects meta-analysis. We also performed additional analysis that stratified treatment options by cytogenetic characteristics. In such analysis, patients were stratified into poor-, intermediate-, and favorable-risk groups by cytogenetic characteristics. OS and RFS benefits of HDAC for different cytogenetic risk groups were analyzed.

## Results

### Studies selected for meta-analysis

**Figure 1:**
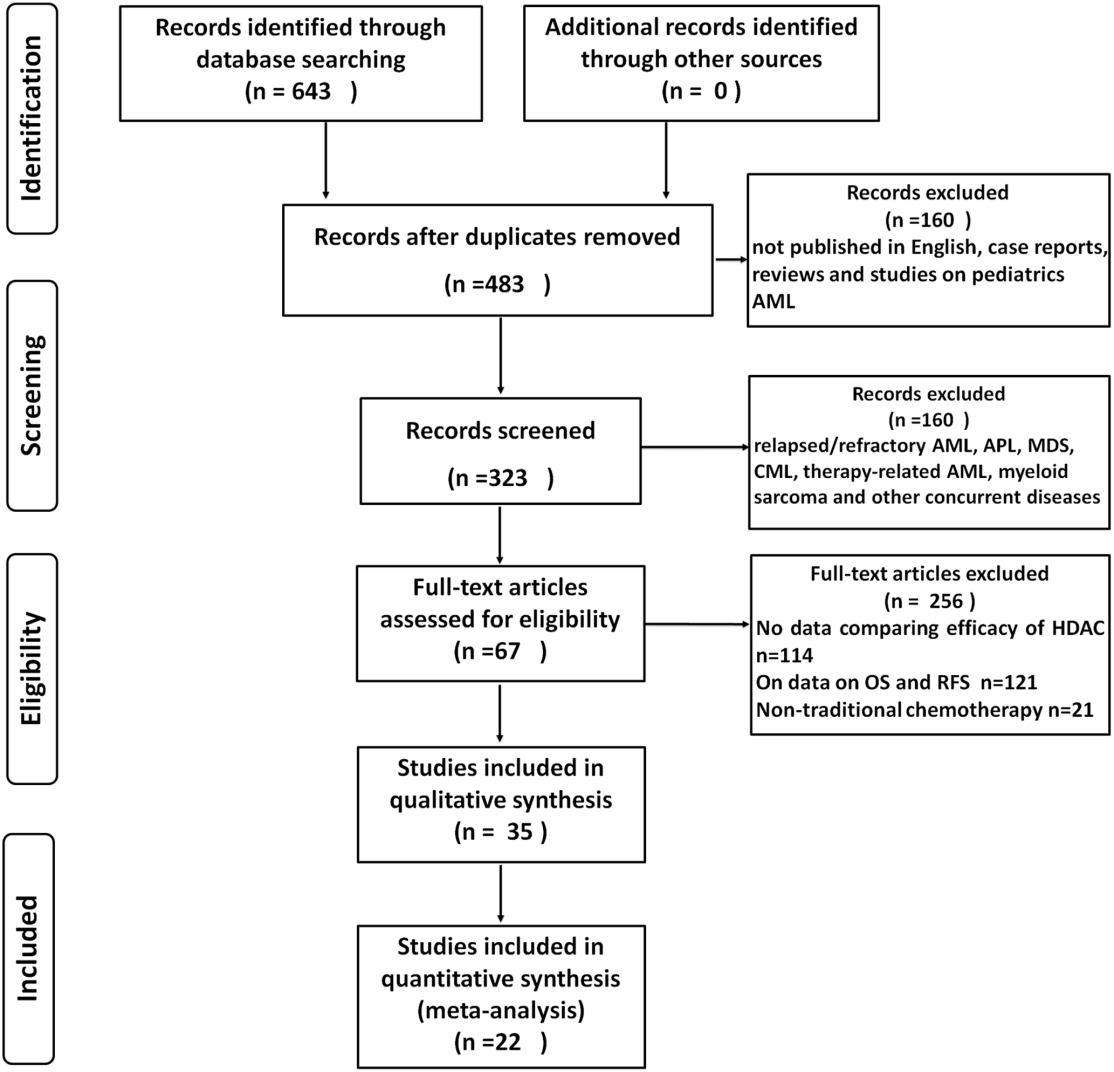
Flow chart explaining the selection of eligible studies included in the meta-analysis.

The initial search on MEDLINE (PubMed) database and the abstract review identified 643 articles. After the screening of titles and abstracts (by two reviewers L.W and G. XY), 160 non-relevant articles were excluded, which were those that were published in languages other than English, case reports, reviews, and studies on pediatrics AML. For the secondary search, the reference lists of review articles were manually examined to identify additional studies. The 483 selected articles were retrieved for further reviews in a structured format. As a result, 160 more articles were excluded, because those studies involved relapsed/refractory AML, APL, high-risk MDS, CML, therapy-related AML, myeloid sarcoma, or other concurrent diseases (including status of other concurrent tumors, definite MDS history) of AML that conflicted with the inclusion criteria. For the remaining 323 articles, full texts were further reviewed. A total of 256 articles were further excluded: 114 articles did not report data comparing the efficacy of HDAC on the OS and RFS of adult AML patients; 121 articles did not provide prospective data on OS and RFS outcome; and 21 articles used non-traditional chemotherapy regimens. The remaining 67 articles met the inclusion criteria. However, 22 articles were further excluded by experts, because the induction or consolidation therapy used in these studies were not consistent, along with confusing risk groups in some articles; 10 more articles were also excluded because only HDAC was used thus no comparison data available; and 4 articles were reporting the same trials [2], [9], [18], [4]. As a result, a total of 22 articles passed through all examinations and were finally used for the meta-analysis in this study [Figure 1].

### Quality Assessment

According to Cochrane methodology, the risk of bias of total RCT articles were assessed by Cochrane factors. The studies at low risk of bias had values (a quantitative index of the risk of bias, range 0–100%) of 64.3%, 64.3%, 0, 92.9%, 50%, 42.9%. (Figure S1).

### HDAC versus SDAC in induction therapy

**Figure 2:**
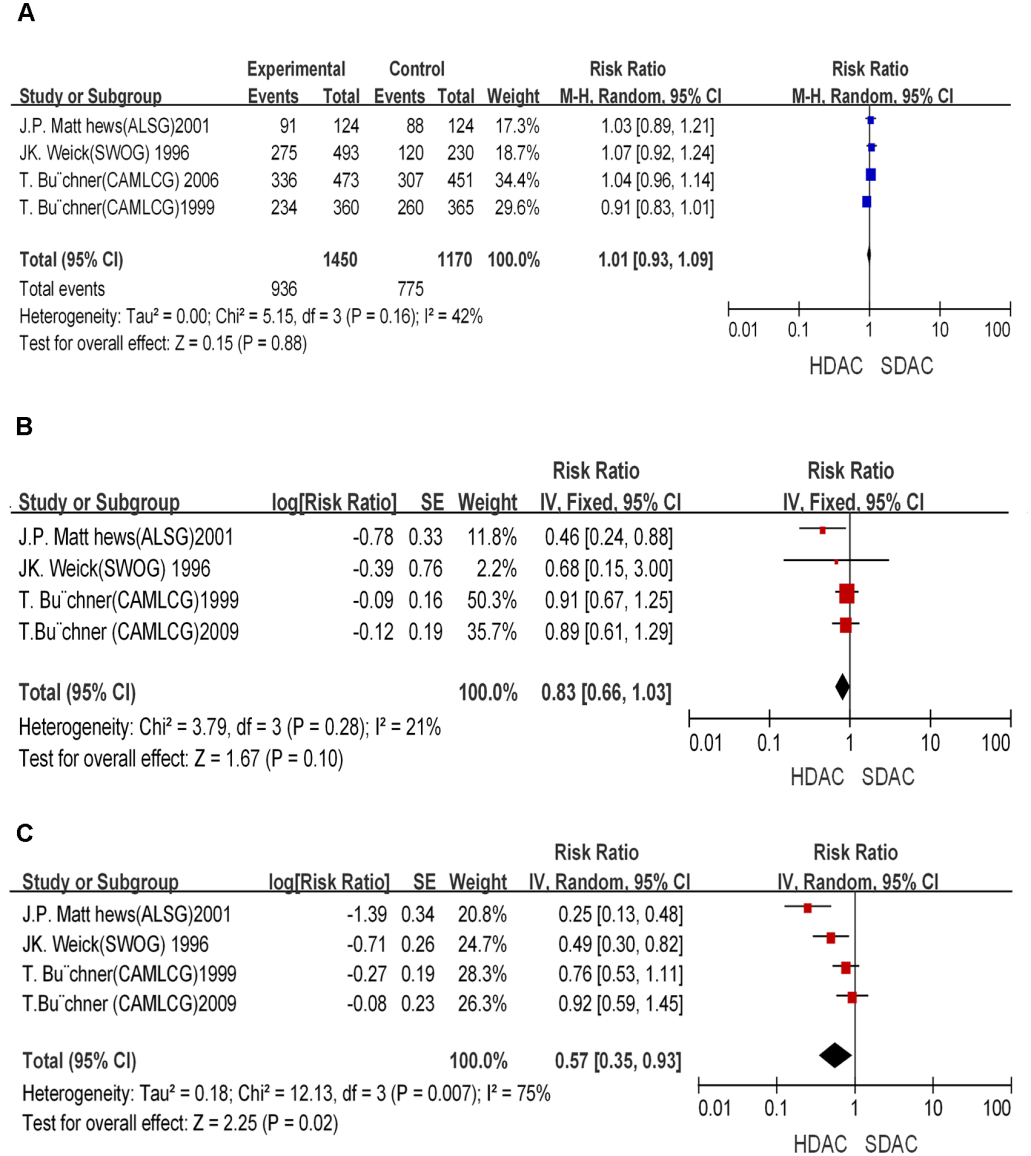
Effect of HDAC versus SDAC in induction therapy. **A**: Effect of HDAC versus SDAC in induction therapy on CR rate. **B**: Overall survival benefit of HDAC in induction therapy. **C**: Relapse free survival benefit of HDAC in induction therapy.

Four randomized controlled trials compared HDAC with SDAC in induction therapy [Table1]. In all 4 trials, the end points of CR, OS, and RFS were reported. Initial baseline characteristics between the treatment group and the control group were quite balanced. A total of 2,980 *de* *novo* AML patients enrolled from 1985 to 2005 were included. In the CAMLCG2009 and CAMLCG 2006 trials, the inclusion criteria for patient age were different from those of the rest of trials. Patients younger than 60 year-old were analyzed in the majority of trials. No significant differences in CR between patients received HDAC and those received SDAC [Figure 2] (HR = 1.01; 95% CI, 0.93–1.09; *P* = 0.88). The OS and RFS results were overall heterogeneous. In the trial ALSG 1996, OS and RFS were much longer in the HDAC group than those in the SDAC group. On the other hand, a larger number of patients receiving HDAC treatment showed shorter OS in the CAMLCG trial. Overall, no significant differences in OS were observed between HDAC and SDAC in the induction phase (HR = 0.83; 95% CI, 0.66–1.03; *P* = 0.1) [Figure 2]. Patients in the HDAC group showed similar OS as that of the SDAC group. However, a statistically significant difference in RFS was observed between HDAC and SDAC in the induction phase (HR = 0.57; 95% CI, 0.35–0.93; *P* = 0.02) [Figure 2]. Therefore, HDAC used in the induction therapy clearly improved RFS but not OS in AML patients.

### HDAC versus SDAC in consolidation therapy

**Figure 3:**
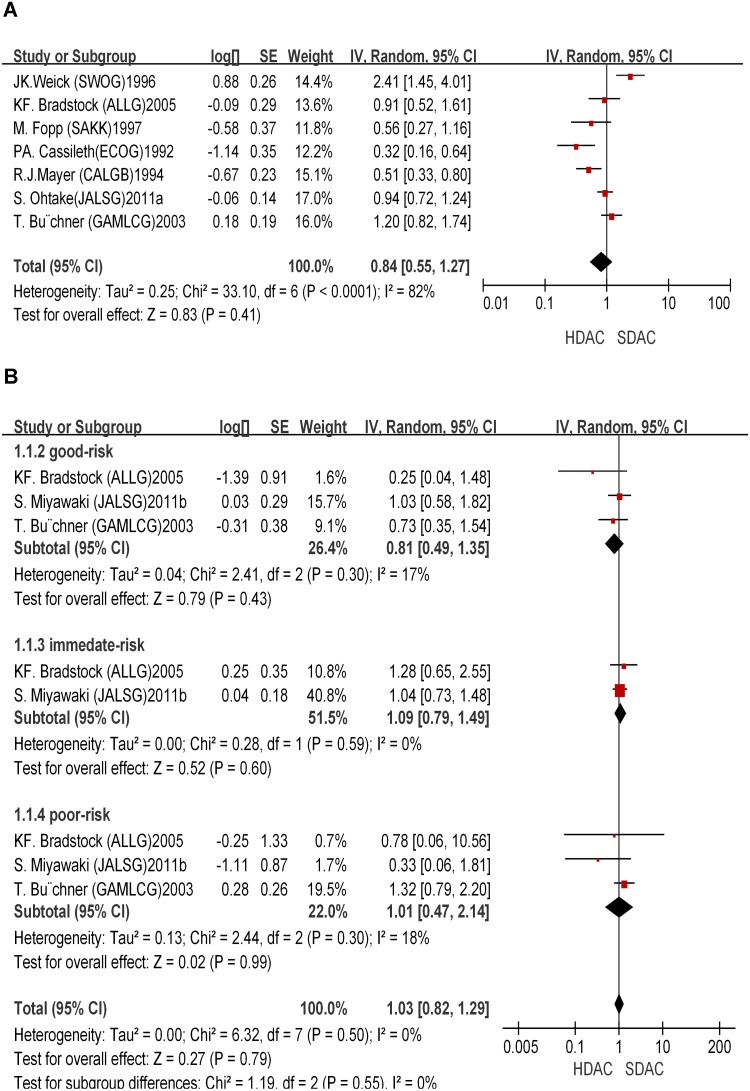
Overall survival benefit of HDAC in consolidation therapy. **A**: Total overall survival benefit of HDAC in consolidation therapy. **B**: Overall survival benefit of different subgroups of HDAC in consolidation therapy.

**Figure 4:**
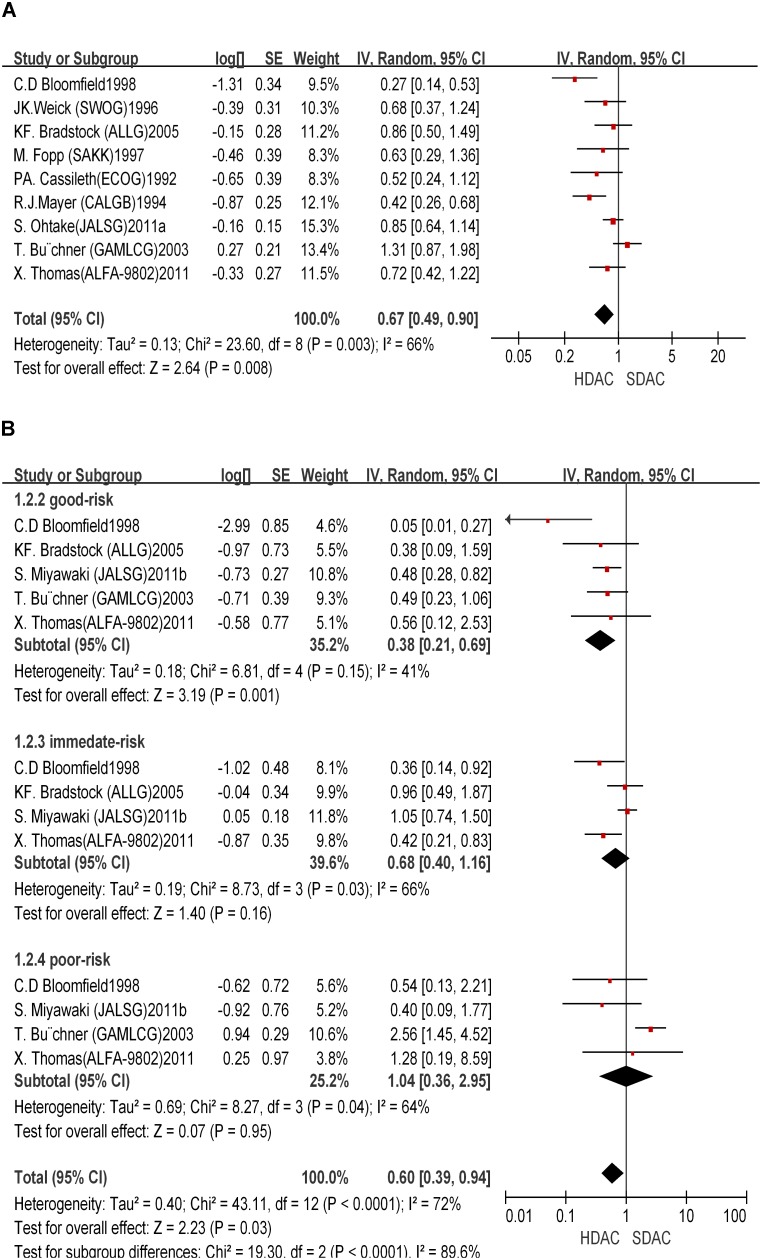
Relapse free survival benefit of HDAC in consolidation therapy. **A**: Total relapse free survival benefit of HDAC in consolidation therapy. **B**: Relapse free survival benefit of different subgroups of HDAC in consolidation therapy.

Nine trials were identified to contain the comparison of HDAC and SDAC in consolidation therapy [Table 2]. All 9 trials were randomized controlled studies, and 7 of them were multicenter trials. A total of 2,965 *de* *novo* AML patients enrolled from 1978 to 2005 were included, and the longest follow-up period of each trial was 10 years. Only patients younger than 60 year-old were analyzed. The initial baseline characteristics (age, sex, race, FAB classification, and cytogenetics) between two groups were similar, although detailed information about initial baseline characteristics in the ECOG1992 trial was not shown. In addition, only 1 course of HDAC was used in the SWOG1996 and SAKK1997 trials, different from all other trials. In 5 trials, HDAC was used concomitantly with other drugs, while 4 other trials only used single dose of Ara-C (2–3 g/m^2^), which may lead to heterogeneity among different trials. All the 9 trials reported end points of 4-year OS and RFS. The 4-year OS rate in the HDAC group ranged from 32%–71%. No significant differences in OS were observed between the HDAC and SDAC groups (HR = 0.84; 95% CI, 0.55–1.27; *P* = 0.41) [Figure 3]. However, patients that used HDAC in consolidation therapy showed longer RFS than those used SDAC (HR = 0.67; 95% CI, 0.49–0.9; *P* = 0.008) [Figure 4]. Therefore, HDAC improved RFS but did not affect OS in consolidation therapy.

**Table 2:** Characteristics of Included Studies for consolidation therapy.

Source	Study ID	EnrollmentPeriod	RCT	Multicenter	No. ofpatients	Median age/agerange (years)	Consolidation therapy	follow-up(years)
JK. Weick et al.1996 [8]	SWOG	1986–1991	Yes	Yes	287	45 (15–64)	DA (DNR+Ara-C 200 mg/m^2^/d×7) continuous 2 coursesDA (DNR+Ara-C 3 g/m^2^ q12 h×6) 1 course	4.3
K.F. Bradstock et al,2005 [12]	ALLG	1995–2000	Yes	Yes	202	43 (15–60)	IcE (IDA+VP-16+Ara-c 100 mg/m^2^/d×5) continuous 2 coursesICE (IDA+VP-16+Ara-c 3 g/m^2^ q12 h d1,3,5,7) 1 course	4
M. Fopp et al,1997 [13]	SAKK	1985–1992	Yes	Yes	137	16–64	DA (DNR+Ara-C 100 mg/m^2^/d×7) 1 coursesDA (DNR+Ara-C 3 g/m^2^ q12 h×3) 1 course	6
PA. Cassileth et al,1992 [14]	ECOG	1984–1988	Yes	Yes	170	15∼65	TA (6-TG+Ara-c 60 mg/m^2^/d×5)AA (Amsa+Ara-c 3 g/m^2^ q12 h×3)(no courses in detail)	4
R.J.Mayer et al,1994 [15]	CALGB	1985–1990	Yes	Yes	389	16–86★	SDAC (Ara-c 100 mg/m^2^/d×5) continuous 4 coursesHDAC (Ara-c 3 g/m^2^ q12 h×3) continuous 4 courses(no detail therapy)	4.3
S. Miyawaki et al,2011b [4]▴	JALSG	2001–2005	Yes	Yes	781	15–64	DA, MA, AA, VEA (DNR, MTZ, Acl-a, VP-16,VCR+Ara-C200 mg/m^2^/dx5) continuous 4 coursesHDAC (2 g/m^2^ q12 h×5) continuous 3 courses	4
S, Ohtake et al,2011a [16]▴	JALSG	2001–2005	Yes	Yes	781	15–64	DA, MA, AA, VEA (DNR, MTZ, Acl-a, VP-16,VCR+Ara-C200 mg/m^2^/dx5) continuous 4 coursesHDAC (2 g/m^2^ q12 h×5) continuous 3 courses	4
T. Büchner et al,2003 [17]	CAMLCG	1992–1999	Yes	Yes	576	16–82★	TAD (6-TG, DNR, Ara-C200 mg/m^2^/dx5)continuous several coursesHAM (MTZ+Ara-c 2 g/m^2^ q12 h d1,2,8,9)	NR
CD. Bloomfield et al,1998 [19]	CALGB	1985–1990	Yes	No	186	>16	Ara-C 100 mg/m^2^/dx5 continuous 4 coursesAra-c 3.0 g/m^2^ q12 h d1,3,5 continuous 4 courses(no detail therapy)	5
X. Thomas et al,2011 [20]	ALFA	1999–2006	Yes	Yes	237	15–50	AA (Amsa+Ara-C),TSC (MTZ+VP-16+Ara-C500 mg/m^2^/dx d8–10)continuous 2 courseAra-c 3.0 g/m^2^ q12 h d1,3,5 continuous 4 courses	10
AM. Tsimberidu et al,2003 [21]	HCG	1996–2000	No	No	120	15–60	Ara-c 3.0 g/m^2^ q12 h d1,3,5 continuous 2 coursespreparative regimen: BU+VP-16+CTX Allo-BMT/auto- SCT	5.3
JL. Harousseau et al,1997 [22]	GOELAM	1987–1994	No	No	517	15–60	ICC (IDR+Ara-c 3.0 g/m^2^ q12 h d1–4) continuous 2 coursespreparative regimen: BU+CTX/TBI+CTX Allo-BMT/auto-SCT	8.5
PA. Cassileth et al,1998 [23]	No	1990–1997	Yes	Yes 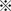	808	16–55	Ara-c 3.0 g/m^2^ q12 h d1–3preparative regimen: CTX+BU Allo-BMT/auto-SCT	4
RA. Zittoun et al,1995 [24]	GIMEMA	1986–1993	Yes	Yes 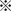	623	33 (10–59)	AA (Amsa+Ara-c 2.0 g/m^2^ d1–6) continuous 2 coursespreparative regimen: CTX+TBI+/−BU Allo-BMT/auto-SCT	8
S. Brunet et al,2004 [25]	Spain	1994–1999	No	No	200	15–60	Ara-c 3.0 g/m^2^ q12 h d1–3 continuous 2 coursespreparative regimen: CTX+TBI+/−BU Allo-BMT/auto-SCT	7
R. Bassan et al,1998 [26]	Italy	1987–1993	No	No	108	15–60	Ara-c 2.0 g/m^2^/d d1–6preparative regimen: Dox+TBI Allo-BMT/auto-SCT	>5
PA. Cassileth et al,1992 [14]	ECOG	---	No	Yes 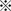	534	44 (15–65)	Ara-c 3.0 g/m^2^ q12 h d1–6 1 coursepreparative regimen: CTX+TBI Allo-BMT	6
GJ. Schiller et al,1992 [27]	----	1982–1990	No	No	103	16–45	MA, DA (MTZ, DNR+Ara-c 2.0–3.0 g/m^2^ q12 h d1–4)2–3 course preparative regimen: TBI+CTX/MTX/Ara-C Allo-BMT	8
JL. Harousseau et al,1991 [28]		1984–1987	No	Yes 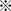	115	44 (13–65)	ICC (Amsa+Ara-c 3.0 g/m^2^ q12 h d1–4)2 course no preparative regimen Allo-BMT	7

Note: ▴ S. Miyawaki et al, 2011 repeated the same trial of S, Ohtake et al, 2011.

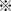
BMT randomized trials were defined that if the patients didn’t have donors, they were randomized into auto-BMT and high-dosed Ara-C groups.

★analyze analyze <60 years the patients in each trial.

Abbreviations: NR, not reported; IDA, idarubicin; Ara-c, cytarabin; VP-16, etoposide; DNR, daunorubicin MCT, multiagent chemotherapy;

CTX, cyclophosphamide; MTZ, mitoxantrone; AZQ, diaziquone; 6-TG, thioguanine; AMS, amsacrine.

We further performed stratified analysis for different subgroups. We restricted the stratification for cytogenetic risk (SWOG/ECOG, NCCN, and MRC) (Table S1). Five trials were included in the stratified analysis. A significant RFS benefit was observed with HDAC treatment (HR = 0.38; 95% CI, 0.21–0.69; *P* = 0.001) in the favorable-risk group [Figure 4]. However, no significant RFS benefit was shown with HDAC treatment in the immediate-risk and poor-risk groups (HR = 0.68; 95% CI, 0.4–1.16; P = 0.16; HR = 1.04; 95% CI, 0.36–2.95; *P* = 0.95). On the contrary, HDAC did not show any significant effects on OS as compared to SDAC. The OS with HDAC was not significantly different from that with SDAC treatment in all 3 stratified risk groups (HR = 0.81; 95% CI, 0.49–1.35; *P* = 0.43; HR = 1.09; 95% CI, 0.79–1.49; *P* = 0.6; HR = 1.01; 95% CI, 0.47–2.14; *P* = 0.99) [Figure 3].

### HDAC versus BMT in consolidation therapy

Nine trials containing the comparison of the effect of HDAC treatment with that of auto-SCT/all-BMT were included in the analysis. They included 5 randomized trials and 4 observational trials. Randomized trials were defined as those in which patients who did not have donors would be randomly allocated into the HDAC and auto-SCT groups. Only 2 trials were multicentre trials. End points of OS and RFS were reported across all cytogenetic risk groups in all 9 trials, so we were not able to perform stratified analysis for different cytogenetic risk groups when evaluating OS and RFS outcomes. A total of 3,128 *de* *novo* AML patients enrolled from 1986 to 2000 were included. The longest follow-up period of each trial was 8.5 years [Table 2]. Of them, 29.8% patients received auto-SCT; 30.8% received allo-BMT; and 39.4% received HDAC. No imbalance in preparative regimen was observed between trials. The data were highly homogeneous in different studies concerning RFS endpoint (*I^2^* = 0%). Only patients younger than 65 year-old were enrolled in the analysis considering the risk of transplantation.

**Figure 5:**
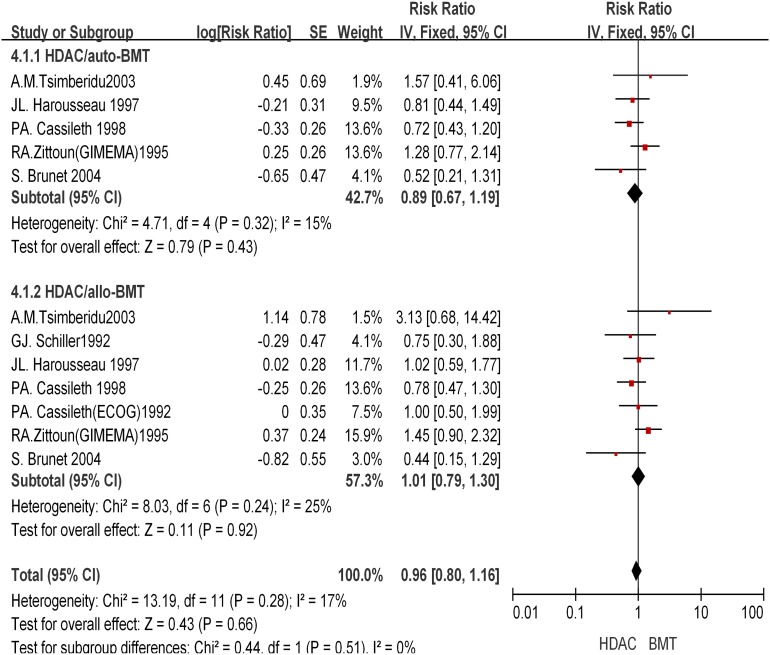
Effect of HDAC versus BMT on overall survival.

**Figure 6:**
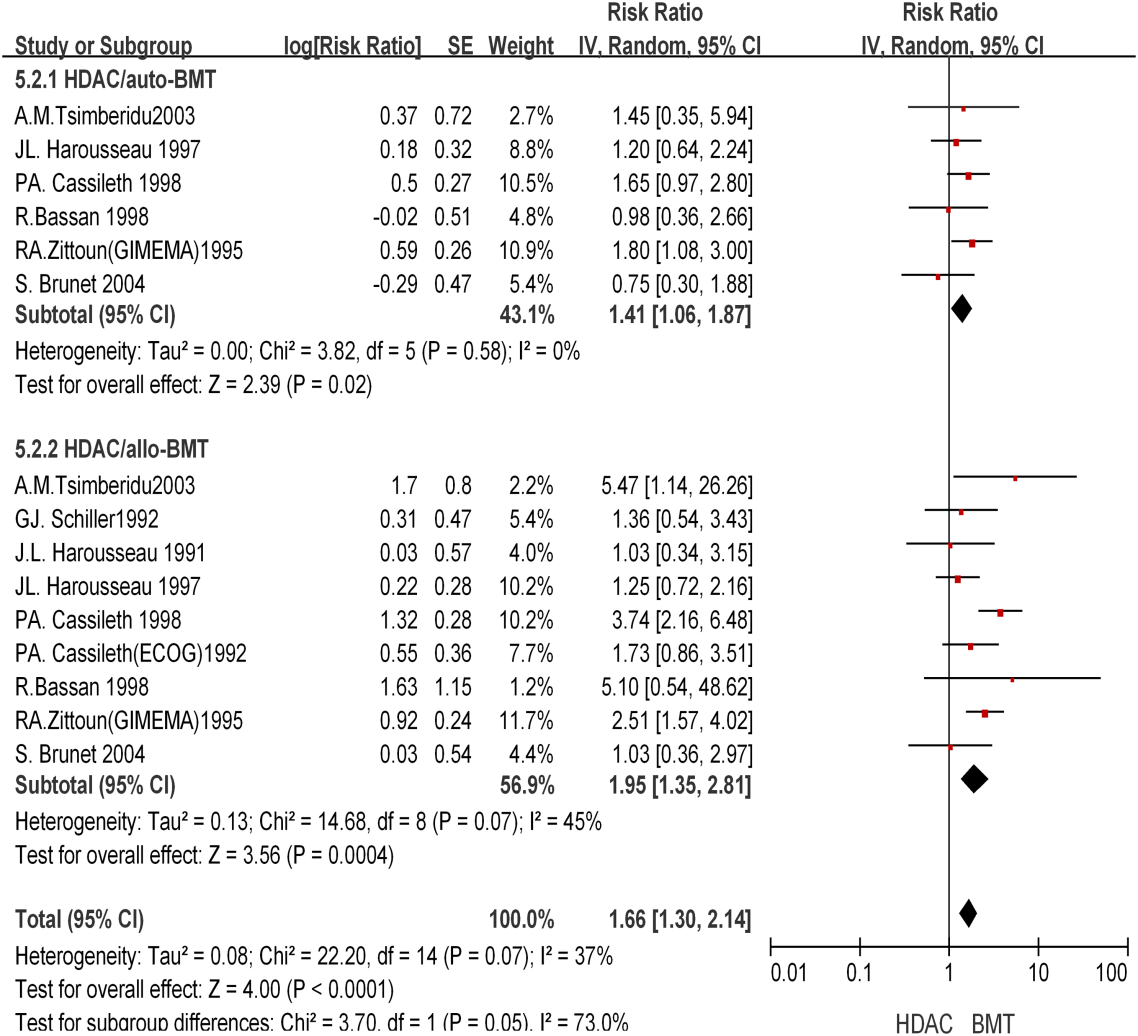
Effect of HDAC versus BMT on relapse free survival.

This analysis revealed that the combined HR was 0.89 (95% CI, 0.67–1.19; *P* = 0.43), 1.01 (95% CI, 0.79–1.3; *P* = 0.92), and that patients received HDAC had an OS similar to that of patients received auto-SCT/allo-BMT in consolidation therapy [Figure 5]. On the other hand, the RFS was significantly different between the auto-SCT/allo-BMT group and the HDAC group [Figure 6]. Auto-SCT had a combined HR of 1.41 (95% CI, 1.06–1.87; *P* = 0.02), while allo-BMT had a combined HR of 1.95 (95% CI, 1.35–2.81; *P* = 0.0004), indicating a significant RFS benefit of auto-SCT/allo-BMT over HDAC. Overall, the results indicated that auto-SCT/allo-BMT significantly reduced the hazard rate of relapse but failed to improve overall survival.

## Discussion

In the past 20 years, Ara-C has been widely used in the induction and consolidation therapy for AML. Multiple prospective studies on Ara-C have been reported, and the application of HDAC has been tested extensively beyond first-line therapy and is considered a standard therapy. However, HDAC started to be questioned in recent studies with larger patient numbers. In this study, we performed a meta-analysis to address whether HDAC application in the induction and consolidation therapy prolongs RFS and decreases AML recurrence comparing with SDAC.

In a recent meta-analysis, 3 trials were analyzed, which discovered no differences in CR rates between HDAC and SDAC treatments. HDAC in induction therapy improved long-term disease control and OS in adults <60 years of age with *de* *novo* AML [29]. However, the effect of HDAC remains unclear in consolidation therapy, especially that for patients younger than 60 years. Therefore, we systematically collected all trials that used HDAC in both induction therapy and consolidation therapy from Jan. 1990 to Mar. 2013. The regimen of induction and consolidation therapy was restricted, which led to the exclusion of 20 articles containing different regimens of induction and consolidation therapy in HDAC and SDAC groups. All trials we identified were reported on an intent-to-treat basis and included a complete description of withdrawals and drop-outs. Some degrees of heterogeneity still existed in the age inclusion criterion. In one article, patients older than 60 years of age were not analyzed separately from patients younger than 60 years. However, this article was still included because the proportion of patients older than 60 years was very low. Based on the current data, we cannot conclude whether HDAC has the same effects on older patients. The dose of HDAC has also been questioned. In HOVON/SAKK study [30], Ara-C was used at 1.0 g/m^2^ q12 h×6 days. In this meta-analysis, we limited HDAC at the dose level of 2.0–3.0 g/m^2^ q12 h×3–5 days for the majority of trials.

Overall, endpoint heterogeneity within trials was limited. No evidence was found to support the notion that HDAC improves CR rate as compared to SDAC in induction therapy. However, our analysis revealed that HDAC had a clear benefit on RFS in induction therapy, consistent to the findings from ALSG and CAMLCG [8], [10]. A retrospective analysis of CALGB and ECOG studies [14], [15], [31] discovered a survival advantage of HDAC in consolidation therapy over SDAC. However, our analysis failed to reach this conclusion. Data from the risk group stratified analysis demonstrated that HDAC significantly improved RFS in the favorable-risk group but no significant benefits in the intermediate and poor-risk groups. We also discussed the advantage of using BMT in consolidation therapy and discovered that auto-BMT/allo-BMT improved RFS, but not OS, as compared to HDAC.

In conclusion, this meta-analysis demonstrated that HDAC improved RFS in induction therapy while reducing the relapse rate in consolidation therapy, as compared with SDAC, especially for the favorable-risk group. Auto-BMT/Allo-BMT had a more beneficial effect in prolonging RFS as compared with HDAC. The analysis also posed some challenges to previous trial results. Overall, treatment with HDAC regimen did show some advantages for some outcome endpoints, especially in certain risk groups. However, it failed to show predominant advantages in all cases. Considering its high toxicity, caution should be taken when HDAC treatment regimen is chosen for patients. We also discovered varied degrees of heterogeneity within trials in our meta-analysis, which may interfere with the interpretation of results and limit the validity of the findings. In the future, more comprehensive clinical trails with improved study designs are needed to help elucidate the advantages and drawbacks of each treatment regimen in order to identify the optimal dose and treatment schedule for AML patients.

## Supporting Information

Checklist S1 PRISMA Checklist.
(DOC)

Figure S1 **Risk of bias graphs of Randomized Control Trials.**
(DOC)

Table S1 Risk status based on validated cytogenetics.
(DOC)
