# Supplementary material for: High-Dose Cytarabine in Acute Myeloid Leukemia Treatment: A Systematic Review and Meta-Analysis
Source: PLoS One. 2014 Oct 9;9(10):e110153. doi: 10.1371/journal.pone.0110153 (PMC4192550; doi:10.1371/journal.pone.0110153)
Supplement: Figure S1 — Risk of bias graphs of Randomized Control Trials. (DOC) [file pone.0110153.s002.doc]

Figure S1：Risk of bias graphs of Randomized Control Trials
