# Supplementary material for: High-Dose Cytarabine in Acute Myeloid Leukemia Treatment: A Systematic Review and Meta-Analysis
Source: PLoS One. 2014 Oct 9;9(10):e110153. doi: 10.1371/journal.pone.0110153 (PMC4192550; doi:10.1371/journal.pone.0110153)
Supplement: Table S1 — Risk status based on validated cytogenetics. (DOC) [file pone.0110153.s003.doc]

Table S1: Risk status based on validated cytogenetics

| Risk status | NCCN | SWOG |
| --- | --- | --- |
| Better-risk | Inv(16),or t(16;16)  t(8;21)  t(15;17) | Inv(16),or t(16;16)  t(8;21)-non 9q-/complex cytogenetics  t(15;17) |
| Intermediate-risk | Normal cytogenetics  +8, alone  t(9;11)  other non-defined | +8, -Y, +6, del(12p)  Normal cytogenetics |
| Poor-risk | Complex  (≥3 clonal chromosomal abnormalities)  Monosomal karyotype  -5,5q-,-7,7q-  11q23-non t(9;11)  Inv(3), t(3;3)  t(6;9)  t(9;22) | Complex  (≥3 clonal chromosomal abnormalities)  -5,5q-,-7,7q-  Inv(3),del(9q), 11q, 20q, 21q,17p  t(6;9), t(9;22)  t(8;21)-with 9q-/complex cytogenetics |
